# Monoclonal Antibody Targeting *Staphylococcus aureus* Surface Protein A (SasA) Protect Against *Staphylococcus aureus* Sepsis and Peritonitis in Mice

**DOI:** 10.1371/journal.pone.0149460

**Published:** 2016-02-29

**Authors:** Yilong Yang, Mengying Qian, Shaoqiong Yi, Shuling Liu, Bing Li, Rui Yu, Qiang Guo, Xiaopeng Zhang, Changming Yu, Jianmin Li, Junjie Xu, Wei Chen

**Affiliations:** 1 Laboratory of Vaccine and Antibody Engineering, Beijing Institute of Biotechnology, Beijing, PR China; 2 Department of Clinical Laboratory, 306 Hospital of People’s Liberation Army, Beijing, PR China; University Medical Center Utrecht, NETHERLANDS

## Abstract

Epidemic methicillin-resistant *Staphylococcus aureus* (MRSA) imposes an increasing impact on public health. Due to multi-antibiotics resistance in MRSA strains, there is an urgent need to develop novel therapeutics such as effective monoclonal antibodies (mAbs) against MRSA infections. *Staphylococcus aureus* surface protein A (SasA), a large surface-located protein (~240 kDa), is one of MSCRAMMs (microbial surface components recognizing adhesive matrix molecules) and a potential target for immunotherapeutic approaches against *S*. *aureus* infections. In the present study, we analyzed the sequence of SasA with bioinformatics tools and generated a protective monoclonal antibody (2H7) targeting the conserved domain of SasA. 2H7 was shown to recognize wild-type *S*. *aureus* and promote opsonophagocytic killing of *S*. *aureus*. In both sepsis and peritoneal infection models, prophylactic administration of 2H7 improved the survival of BALB/c mice challenged by *S*. *aureus* strain USA300 and ST239 (prevalent MRSA clones in North America and Asian countries, respectively) and enhanced bacterial clearance in kidneys. Additionally, 2H7 prophylaxis prevented the formation of intraperitoneal abscess in a murine model of peritoneal infection and therapeutic administration of 2H7 showed protective efficacy in a murine sepsis model. Our results presented here provide supporting evidences that an anti-SasA mAb might be a potential component in an antibody-based immunotherapeutic treatment of MRSA infections.

## Introduction

*Staphylococcus aureus*, one of the most important human pathogens, causes a variety of infections, including skin, soft tissue, and bone infections and life-threatening sepsis, endocarditis and pneumonia [1]. The high proportions of methicillin-resistant *S*. *aureus* (MRSA) indicate an increased public health risk and a need for second-line antibiotics, which increase costs and exert problematic side effects [2–4]. Therefore, the development of an alternative treatment option for *S*. *aureus* infections, particularly immunotherapeutic approaches, is being extensively investigated. One such strategy is to develop effective monoclonal antibodies (mAbs) against MRSA infections [5,6].

Many antigens of *S*. *aureus* have been tested as candidate targets for mAbs against *S*. *aureus* infections, including secreted toxins [7–10], microbial surface components recognizing adhesive matrix molecules (MSCRAMMs) [11–17], lipoteichoic acid (LTA) [18] and quorum sensing peptide [19]. *S*. *aureus* surface protein A (SasA), one of MSCRAMMs, is also known as Srap (serine-rich adhesin for platelets) as it mediates the direct binding of *S*. *aureus* to platelets and contributes to infective endocarditis [20]. SasA is composed of 2,271 amino acids and belongs to the serine-rich repeat proteins (SRRPs) family [21]. Consistent with a previous report that SRRPs are potential vaccine candidates [21], active immunization with SasA can induce a specific antibody response and protect BALB/c mice against staphylococcal lethal challenge [22]. Of note, SasA is prevalent among clinical *S*. *aureus* strains. In one study, the SasA gene was present in 100% of disease isolates (n = 155) and carriage isolates (n = 179) [23]. Additionally, by bioinformatics analysis, we showed here that there exists a conserved region of SasA among a diverse range of clinical *S*. *aureus* strains. Moreover, SasA is expressed during *S*. *aureus* infections in vivo. IgG titers against SasA in serum obtained from patients convalescing from *S*. *aureus* infections were higher than those obtained from healthy individuals [23]. Taken together, we envision that SasA can be regarded as a potential target for a mAb-based immunotherapy against *S*. *aureus* infections.

In this study, we analyzed the sequence of SasA with bioinformatics tools and generated a mAb targeting the conserved region of SasA. This mAb (2H7) was characterized in vitro and its protective efficacy was evaluated in vivo. Passive immunization with a single dose of 2H7 conferred protection against murine sepsis and peritonitis caused by MRSA challenges. Data presented here imply that an anti-SasA mAb might be a potential component in an antibody-based immunotherapeutic treatment of MRSA infections.

## Materials and Methods

### Ethics statement

All of the animal experiments in this study were approved by the Laboratory Animal Care and Use Committee of the Beijing Institute of Biotechnology (Beijing; Permit Number 2015–02). All surgery was performed under sodium pentobarbital anesthesia and mice were sacrificed at indicated time by CO_2_ inhalation. All efforts were made to minimize suffering.

### Mice

Specific-pathogen-free (SPF) BALB/c mice (female, 5-week-old) were purchased from Vital River Laboratory Animal Technology Co. Ltd. (Beijing, China), housed under controlled ambient conditions (12 h light/dark cycle), and fed laboratory chow and distilled water *ad libitum*. Mice were allowed to acclimatize for a period of 7 days before the corresponding experiment.

### Bacteria

*S*. *aureus* USA300 FPR3757 (BAA-1556™) was obtained from American Type Culture Collection (Manassas, VA, USA). USA300 is one of the most prevalent MRSA clones in North America and Europe [24]. *S*. *aureus* strain ST239, isolated in 2013 from a patient hospitalized with pneumonia in the 306 Hospital of People’s Liberation Army (Beijing, China), is a clinical MRSA strain and belongs to multilocus sequence type 239. ST239 is the predominant hospital-acquired MRSA clone in Asian countries [25]. Overnight cultures of Staphylococci were diluted 1:100 into tryptic soy broth (TSB) and grown to OD_600_ ~0.8 at 37°C. PBS-washed Staphylococci were adjusted to an appropriate concentration.

### Recombinant proteins

The SRR1 region (48–229 aa), NRR region (230–751 aa), NRR1 region (230–540 aa), NRR2 region (490–751 aa) and SRR1-NRR1 region (48–540 aa) of the full-length SasA were amplified from USA300 FPR3757 (BAA-1556™) genomic DNA by PCR. The primers used in this study are listed in S1 Table. The PCR products were cloned into *Escherichia coli* vector pET21a (+) (Novagen) and transformed into *E*. *coli* strain BL21 (DE3) for the expression of recombinant fusion proteins containing an N terminal six-histidine-residue tag. The purified proteins were isolated by Q Sepharose Fast Flow and HisTrap HP (GE Healthcare) chromatography.

### Preparation of monoclonal antibodies

The murine monoclonal antibodies targeting SRR1-NRR1 (48–540 aa) were generated by the standard hybridoma method described in earlier studies [26], followed by enzyme-linked immunosorbent assay (ELISA) and western blot screening. Antibodies were purified by protein G affinity chromatography. IgG subclass was identified using the Rapid Antibody Isotyping Kit (Thermo Scientific).

### Enzyme-linked immunosorbent assay (ELISA)

The purified antigens (SRR1, NRR, NRR1, NRR2 and SRR1-NRR1) were coated onto microplates (96-well) in 0.1 M carbonate buffer (pH 9.6) at a concentration of 2 μg/ml overnight at 4°C. The plates were blocked with 5% nonfat milk and incubated with serial dilutions of mAbs for 1 h at 37°C. The secondary anti-mouse IgG HRP-linked antibody (Cell Signaling) was then applied for an additional hour at 37°C. The plates were washed and incubated with 3, 3’, 5, 5’-tetramethylbenzidine dihydrochloride (Sigma) as the substrate. The A450 was measured using a Spectra Max photometer (Molecular Devices). To detect the binding of SasA mAb to wild-type *S*. *aureus*, the formalin-fixed USA300 was diluted in PBS containing 0.25% glutaraldehyde to an optical density (600 nm) of 0.15 and coated onto microplates. Rabbit serum (1%) was added to reduce the non-specific interactions between murine mAbs and protein A expressed on *S*. *aureus*.

### Mouse platelets binding assay

Purified SRR1 and NRR were conjugated with Cy3 (GE Healthcare). Formalin-fixed Mouse platelets were prepared as described previously [20]. To measure the binding of SRR1 and NRR to platelets, 10^5^ fixed mouse platelets were incubated with FITC-labeled anti CD-41 antibody (BioLegend) and increasing concentrations of SRR1-Cy3 or NRR-Cy3 (20 nM, 100 nM and 500 nM) at room temperature for 1 h. For the binding competition assay, 2000 nM NRR was mixed with 100 nM NRR-Cy3 to test whether the binding of NRR-Cy3 to mouse platelets was affected. Additionally, 2000 nM 2H7 or control mAb was incubated with 100 nM NRR-Cy3 overnight at 4°C to examine whether 2H7 could inhibit the association between NRR-Cy3 and mouse platelets. The samples were analyzed by flow cytometry (BD FACSCalibur).

### Opsonophagocytic killing assay

In the ex vivo whole-blood killing model [15], blood was taken from anesthetized BALB/c mice (female, 6-week-old) by cardiac puncture and collected in a vial containing Heparin (Sigma). PBS-washed USA300 was adjusted to a concentration of 3×10^5^ CFU/ml. Then, 100 μl of *S*. *aureus* was mixed with 200 μl of pooled mouse blood containing 10 μg/ml 2H7 or isotype control antibody. In addition, a control experiment was conducted to address the possibility that antibodies mediate bacterial aggregations and result in a drop in apparent CFUs. Blood cells were first lysed in the presence of 0.5% saponin and further incubated with USA300 and antibodies as described above.

Opsonophagocytic killing with human neutrophils was conducted as follows. HL60 cells (ATCC) were cultured, differentiated to granulocytes and washed as described previously [27]. In 5 to 6 days, 80 to 85% of *N*, *N*-dimethyl- formamide-induced HL60 cells were differentiated into granulocytes. Guinea pig serum was used as complement source. To remove the cross-reactive antibodies, serum was incubated with *S*. *aureus* USA300 (4°C for 30 min), centrifuged and filter-sterilized. The assay was performed in a 96-well plate containing 1×10^4^ CFU USA300, 1×10^5^ viable differentiated-HL60 cells, 4% complement serum and 10 μg/ml 2H7 or isotype control antibody. The mixed samples were incubated at 37°C with shaking. At 0 and 60 min, 10 μl aliquots were removed and diluted with 0.5% saponin-PBS. Then, the samples were sonicated briefly to disrupt bacterial aggregates and plated on TSA to determine the viable CFU numbers. The relative killing was calculated as the percent difference in CFU between samples at 0 min and 60 min.

### Intravenous challenge model

For the prophylaxis studies, female BALB/c mice (6-week-old, n = 10) were injected intraperitoneally with a single dose of 2H7 (at 5, 15 or 25 mg/kg) or isotype control mAb (15 mg/kg) 24 h prior to challenge by intravenous injection with 1×10^8^ colony-forming units (CFUs) of USA300 or 2×10^8^ CFUs of ST239. For the therapeutic studies, 1×10^8^ CFUs of USA300 was delivered intravenously 1 h or 3 h prior to intraperitoneal injection with 2H7 or control antibody (15 mg/kg). The challenged mice were monitored over a period of 9 days.

To examine if 2H7 can reduce the staphylococcal burden in organs, female BALB/c mice (6-week-old, n = 9) were passively immunized with 2H7 or isotype control mAb (15 mg/kg) 24 h prior to challenge by intravenous injection with 1×10^7^ CFUs of USA300. On day 4 following the challenge, the infected mice were euthanized by CO_2_ inhalation. Both kidneys were harvested and homogenized. The samples were sonicated briefly to disrupt bacterial aggregates and plated on tryptic soy agar for CFU enumeration.

### Intraperitoneal challenge model

For the lethal challenge studies, female BALB/c mice (6-week-old, n = 10) were injected intraperitoneally with a single dose (at 5, 15 or 25 mg/kg) of 2H7 or isotype control mAb (15 mg/kg) 24 h prior to challenge by intraperitoneal injection with 2×10^9^ CFUs of USA300 or 4×10^9^ CFUs of ST239. The challenged mice were monitored over a period of 7 days.

For the sublethal challenge studies, female BALB/c mice (6-week-old, n = 9–10) were passively immunized with 2H7 (15 mg/kg) or isotype control mAb (15 mg/kg) 24 h prior to challenge by intraperitoneal injection with 1×10^9^ CFUs of USA300. On day 2 following the challenge, the infected mice were euthanized by CO_2_ inhalation and necropsied. A person blinded to the identity of the mouse groups examined the presence of peritoneal abscesses, which can be observed by the naked eye. As defined in earlier studies [8], the peritoneal abscesses were yellow, round or oval (diameter about 2–4 mm), and raised staphylococcal lesions located on the anterior abdominal wall at the inoculation site. The peritoneal abscesses were not detected in uninfected mice. Both kidneys were also harvested and homogenized. The samples were sonicated briefly to disrupt bacterial aggregates and plated on tryptic soy agar for CFU enumeration.

### Mice euthanasia

In the survival study, although mice died as a direct result of the experimental intervention, we used humane endpoints and euthanized mice displaying severe illness prior to the end of our experiments to minimize pain and distress. Following challenges, the conditions of mice were monitored every 8 hours for a period of 9 days (intravenous challenge model) or 7 days (intraperitoneal challenge model). Any mice displaying severe illness as determined by weight loss of greater than 20%, a hunched posture, loss of ability to ambulate, labored breathing, and ruffled fur were euthanized by CO_2_ inhalation. At the completion of all animal experiments, survivors were euthanized by CO_2_ exposure in accordance with IACUC policy. Death was verified by monitoring cardiac cessation and respiratory arrest. There was no unexpected death in this study.

### Statistical analysis

A two-tailed unpaired t test was performed to measure the statistical significance of the platelets-binding data, the phagocytotic killing of *S*. *aureus* and the bacterial loads in the kidney. The survival curves were analyzed by the log-rank Mantel-Cox test. The formation of peritoneal abscesses was analyzed through the Chi-square with Yates' correction test (two-sided). Probability (P) value ≤ 0.05 was considered significant. All of the statistical data were analyzed using GraphPad Prism.

## Results

### Sequence analysis of SasA

**Fig 1:**
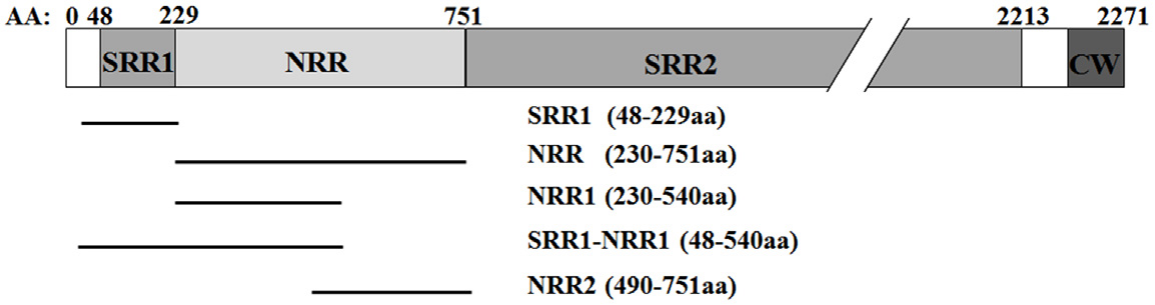
Sequence analysis of SasA. The full-length amino acid sequence of SasA was analyzed using bioinformatics tools. SasA is mainly composed of a short serine-rich repeat region (SRR1), a non-repeat region (NRR), a second long serine-rich repeat region (SRR2) and a C-terminal cell wall anchor motif (CW). Sequence homology analysis showed that NRR is the most conserved region of SasA. Different regions of SasA were expressed and purified. These are indicated below the full-length SasA.

SasA is a high-molecular-weight protein (~240 kDa) and the difficulties in construction of full-length recombinant SasA limit its immunotherapeutic application. Therefore, sequence analysis of SasA is an essential step for the development of protective mAbs. We screened 36 whole-genome sequences of *S*. *aureus* strains that are publicly available. These strains can be classified into 19 different sequence types and represent a diverse range of *S*. *aureus* clonal population (S1 Fig and S2 Table). We found that all of these strains carry the SasA gene. We analyzed the full-length amino acid sequence of SasA (*S*. *aureus* USA300 FPR3757) with bioinformatics tools (Motif scan, http://myhits.isb-sib.ch/cgi-bin/motif_scan). Two serine-rich repeat regions (SRR) were found, and a non-repeat region (NRR) was found between these SRRs. The amino acid sequences of SRR1, NRR and SRR2 represent 48–229 aa, 230–751 aa and 752–2213 aa of full-length SasA, respectively (Fig 1). Next, we conducted the sequence homology analysis by using a multi-alignment method [28]. SRR1, NRR and SRR2 display 75.8%, 90.4% and 49.3% identity at the amino acid level, respectively, suggesting that NRR is the most conserved region of SasA (S2 Table). Moreover, we prepared different recombinant fragments of SasA (Fig 1) and showed that immunization with SRR1 (48–229 aa) and NRR1 (230–540 aa) generated protective immunity against lethal infection with *S*. *aureus* (S2 Fig).

### Monoclonal antibodies to SasA

According to other SRRPs, the main function of the SRR2 region is supposed to extend the SRR1 and NRR regions to the bacterial surface, where SRR1 and NRR can mediate adherence of bacteria to host tissue [21,29]. Thus, SRR1 and NRR may be more accessible to immune cells in vivo [21]. Based on the overall structure, conserved region and immunoprotective domain of SasA, we chose the SRR1-NRR1 region (48–540 aa) as the immunogen and generated mAbs by using hybridoma technology. mAbs targeting different epitopes of SasA were screened using the ELISA assays as well as in vivo experiments (S3 and S4 Tables). 2H7 was selected for further study, as 2H7 displayed the best protection in a murine *S*. *aureus* challenge model (S4 Table). 2H7 was determined to belong to the IgG1 subclass.

### 2H7 recognizes the conserved domain of SasA and binds to *S*. *aureus*

**Fig 2:**
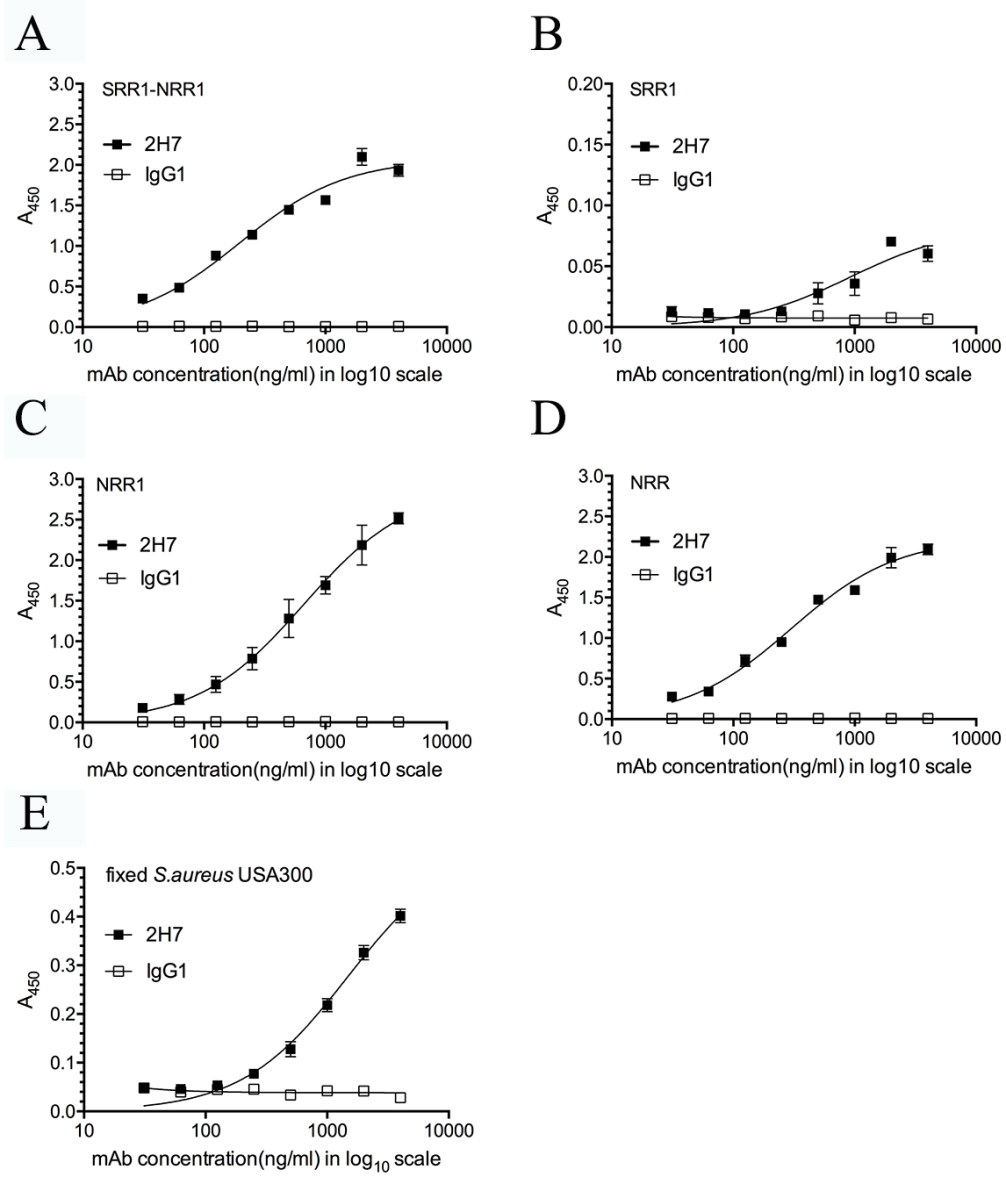
2H7 recognizes the conserved domain of SasA and binds to *S*. *aureus*. ELISA was used to determine the concentration-dependent binding of 2H7 and an isotype control mAb to SasA or *S*. *aureus* USA300. (A to D) ELISA plates were coated with purified antigens (SRR1, NRR, NRR1, NRR2 and SRR1-NRR1) and blocked with 5% nonfat milk. (E) ELISA plate was coated with formalin-fixed *S*. *aureus* USA300. Rabbit serum (1%) was added to block the surface expressed protein A of *S*. *aureus*. Each point represents the antibody concentration and its absorbance at 450 nm. The data represent the means ± SEM (n = 3).

Microplates were coated with different recombinant fragments of SasA (SRR1-NRR1, SRR1, NRR1 and NRR), and ELISA was used to determine the binding activity of 2H7 to SasA. The data showed that 2H7 did not recognize SRR1 but bound with high affinity to NRR1 and NRR (Fig 2 and S3 Table), indicating that 2H7 recognizes the conserved domain of SasA. When bacteria were used as the antigen in the ELISA assay, 2H7 also displayed the binding activity to native SasA expressed on the surface of wild-type *S*. *aureus* (Fig 2E).

### Lack of effect of 2H7 on the binding of SasA to mouse platelets

**Fig 3:**
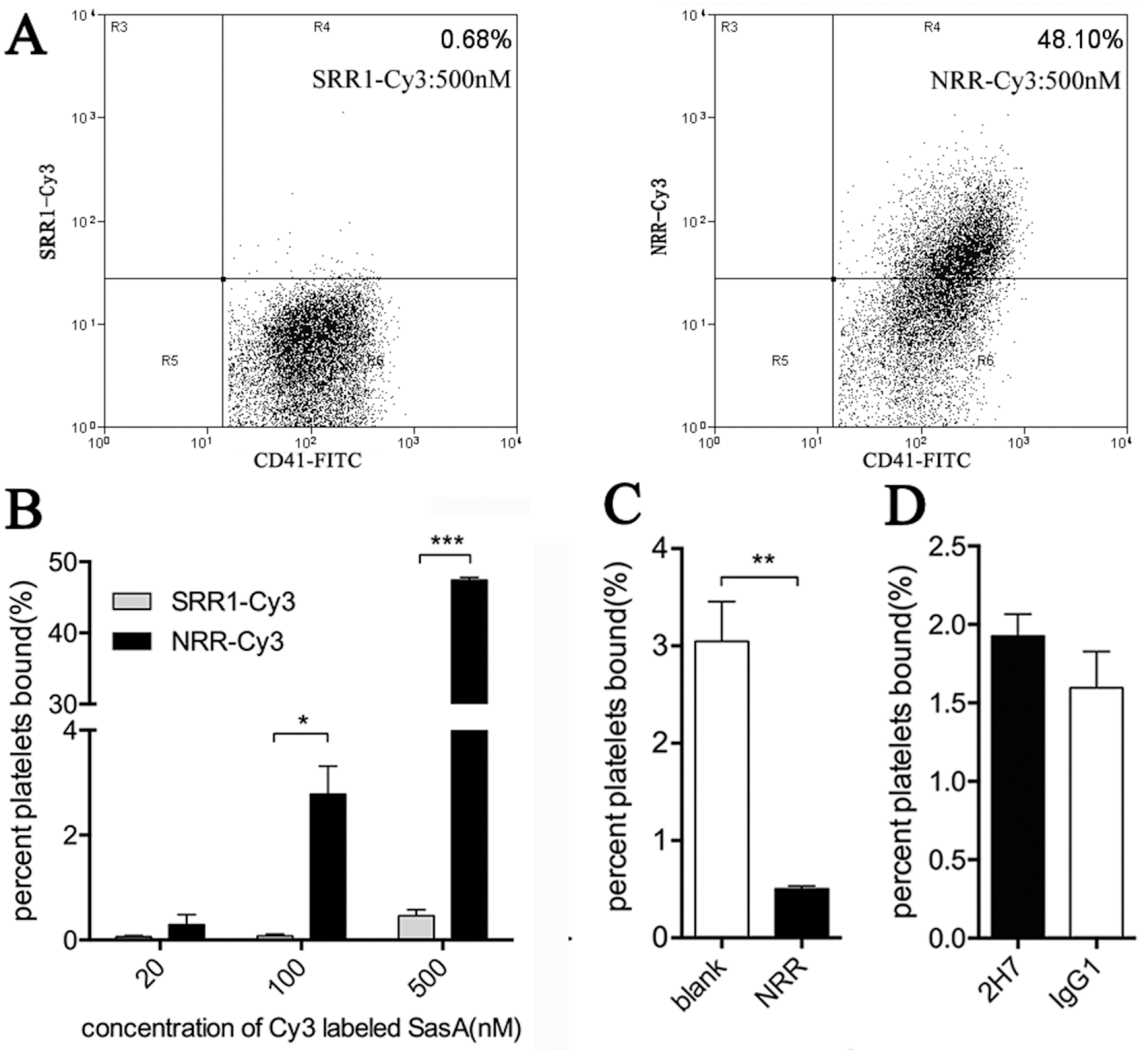
Lack of effect of 2H7 on the binding of SasA to mouse platelets. Fixed mouse platelets were incubated with FITC-labeled anti-CD-41 antibody and Cy3-labeled SasA at room temperature for 1 h and analyzed by flow cytometry. (A) Representative flow-cytometric graph of the binding of Cy3-labeled SRR1 (500 nM) or NRR (500 nM) to mouse platelets. (B) Fixed mouse platelets were incubated with SRR1-Cy3 or NRR-Cy3 (20 nM, 100 nM and 500 nM). (C) Fixed mouse platelets were incubated with NRR-Cy3 (100 nM) in the presence of NRR (2000 nM). (D) NRR-Cy3 (100 nM) was incubated with 2H7 or an isotype control mAb (2000 nM) overnight at 4°C before the incubation with mouse platelets. The data shown in panels B to D represent the means ± SEM (n = 3). The statistical significance was measured using a two-tailed unpaired t test (*: p<0.05, **: p<0.01, ***: p<0.001).

A previous research study showed that SasA (90–723 aa) can bind to human platelets [20]. The interaction between *S*. *aureus* and platelets may play a pathogenetic role when *S*. *aureus* invade the bloodstream. First, we confirmed that SasA was also able to bind to mouse platelets. Compared with Cy3-labeled SRR1 (SRR1-Cy3), Cy3-labeled NRR (NRR-Cy3) bound directly to mouse platelets in a dose-dependent manner (Fig 3A and 3B), and NRR interfered with the binding of NRR-Cy3 to mouse platelets (p = 0.0035) (Fig 3C). Next, we examined the possibility that 2H7 might inhibit the binding of SasA to mouse platelets. However, the addition of SasA mAb 2H7 did not interfere with the association between NRR-Cy3 and mouse platelets (p = 0.289) (Fig 3D).

### 2H7 promotes opsonophagocytic killing of *S*. *aureus*

**Fig 4:**
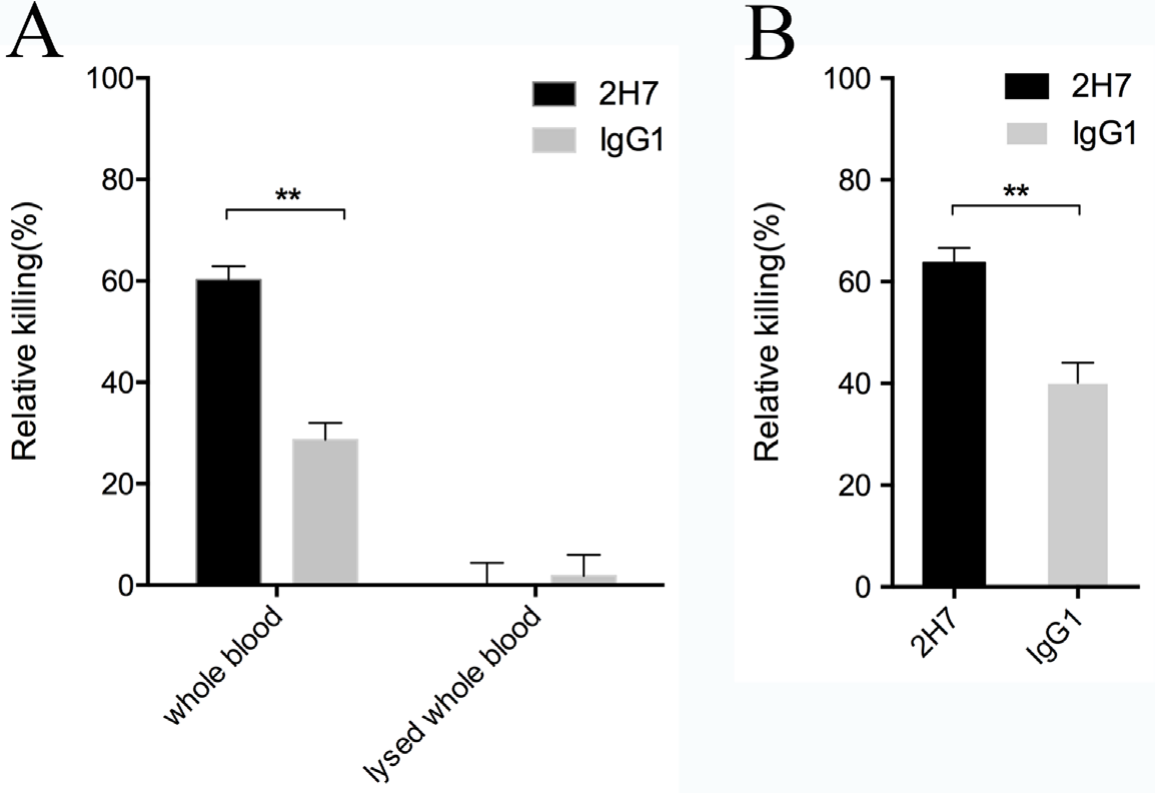
2H7 promotes opsonophagocytic killing by mouse whole blood and human neutrophils. (A) PBS-washed USA300 (3×10^4^ CFU) was incubated with 200 μl heparinized whole blood collected from BALB/c mice (female, 6-week-old) in the presence of 10 μg/ml 2H7 or isotype control antibody. In addition, blood cells were first lysed in the presence of 0.5% saponin and a control experiment was conducted to examine if 2H7 mediate bacterial aggregations and result in a drop in apparent CFUs. (B) HL60 cells were cultured and differentiated to granulocytes. PBS-washed USA300 (1×10^4^ CFU) was incubated with viable differentiated-HL60 cells (1×10^5^), 4% Guinea pig serum (the cross-reactive antibodies were removed) and 10 μg/ml 2H7 or isotype control antibody. The samples were incubated at 37°C with shaking for 60 minutes. The CFUs of *S*. *aureus* were determined by plating on TSA. The relative killing was calculated as the percent difference in CFU between samples at 0 min and 60 min. The data represent the means ± SEM (n = 3). The statistical significance was measured using a two-tailed unpaired t test (**: p<0.01).

Neutrophils play an important role in the front-line of host-pathogen battle. Antibodies function as an opsonin and promote the phagocytosis of pathogen by neutrophils in blood. As 2H7 can recognize SasA expressed on the surface of *S*. *aureus*, we tested whether 2H7 could promote the opsonophagocytic killing of *S*. *aureus*. In the ex vivo whole-blood killing model [15], 2H7 significantly enhanced bacterial killing by mouse whole blood compared with the IgG1 control antibody (relative killing, 60% ± 2% and 29% ± 3%, respectively; p = 0.0017) (Fig 4A). We excluded the possibility that antibodies mediate bacterial aggregations and result in the drop in CFUs. Without functional neutrophils, a reduction in CFUs of *S*. *aureus* was not observed (Fig 4A). In addition, 2H7 was able to promote opsonophagocytic killing of *S*. *aureus* by human neutrophils HL-60 compared with the IgG1 control antibody (relative killing, 64% ± 2% and 40% ± 2%, respectively; p = 0.0011) (Fig 4B).

### In vivo protection against lethal challenges with MRSA in a sepsis model

**Fig 5:**
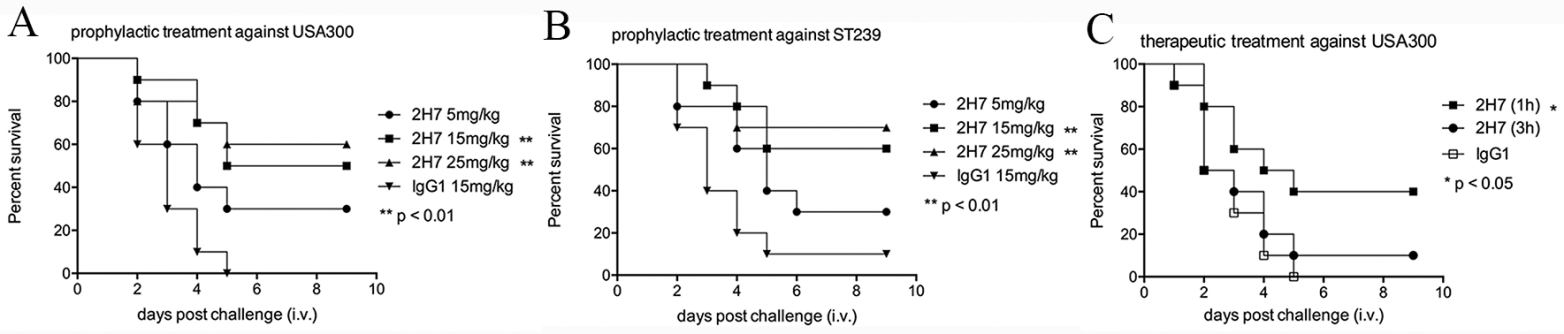
2H7 conferred protection against lethal challenges with MRSA in a sepsis model. (A to B) For the prophylaxis studies, female BALB/c mice (6-week-old, n = 10) were injected intraperitoneally with a single dose of 2H7 (at 5, 15 or 25 mg/kg) or isotype control mAb (15 mg/kg) 24 h prior to challenge by intravenous injection with 1×10^8^ CFUs of USA300 (A) or 2×10^8^ CFUs of ST239 (B). (C) For the therapeutic studies, female BALB/c mice (6-week-old, n = 10) were challenged by intravenous injection with 1×10^8^ CFUs of USA300 1 h or 3 h prior to intraperitoneal injection of 2H7 or control antibody (15 mg/kg). The challenged mice were monitored over a period of 9 days. The survival curves were analyzed by the log-rank Mantel-Cox test (**: p<0.01, *: p<0.05). i.v., intravenous.

Effective monoclonal antibodies should confer protection against lethal challenges. In a murine sepsis model, we evaluated the ability of 2H7 to provide protection against challenge with a lethal dose of *S*. *aureus* strain USA300. USA300 is one of the most frequent sources of community-acquired MRSA infections in the United States, Canada and Europe [24]. Thus protection against USA300 is clinically meaningful. BALB/c mice (n = 10) were passively immunized with 2H7 (at 5, 15 or 25 mg/kg) or isotype control mAb IgG1 (15 mg/kg). Twenty-four hours following passive immunization, 1×10^8^ CFUs of USA300 were administered intravenously. All of the control antibody-treated mice died within 5 days (Fig 5A). In contrast, mice that received a single dose of 2H7 (15 mg/kg or 25 mg/kg) had a higher survival rate (survival rate is 50% and 60%, respectively) (Fig 5A). The significance of protection was analyzed by the log-rank Mantel-Cox test (15 mg/kg, p = 0.0014; 25 mg/kg, p = 0.0027). Passive immunization with a low dose of 2H7 (5 mg/kg) was not able to provide protection (p = 0.060).

Protection against different clinical strains is readily appreciated for development of protective antibodies. Thus we tested if 2H7 can protect against another clinical *S*. *aureus* strain, ST239, the predominant hospital-acquired MRSA clone in Asian countries [25]. Compared with treatment with isotype control antibody (15 mg/kg), treatment with a medium dose (15 mg/kg) or high dose (25 mg/kg) of 2H7 led to significant protection against challenges with 2×10^8^ CFUs of ST239 (15 mg/kg, the survival rate is 60%, p = 0.0049; 25 mg/kg, the survival rate is 70%, p = 0.0035) (Fig 5B).

To examine the therapeutic potential of 2H7, a single dose (15 mg/kg) of 2H7 was administrated 1 h post challenge with 1×10^8^ CFUs of USA300. 2H7 protected 40% of mice from death (p = 0.023). However, we could not extend the therapeutic window beyond 3 h. (Fig 5C)

### In vivo protection against challenges with MRSA in a peritoneal infection model

**Fig 6:**
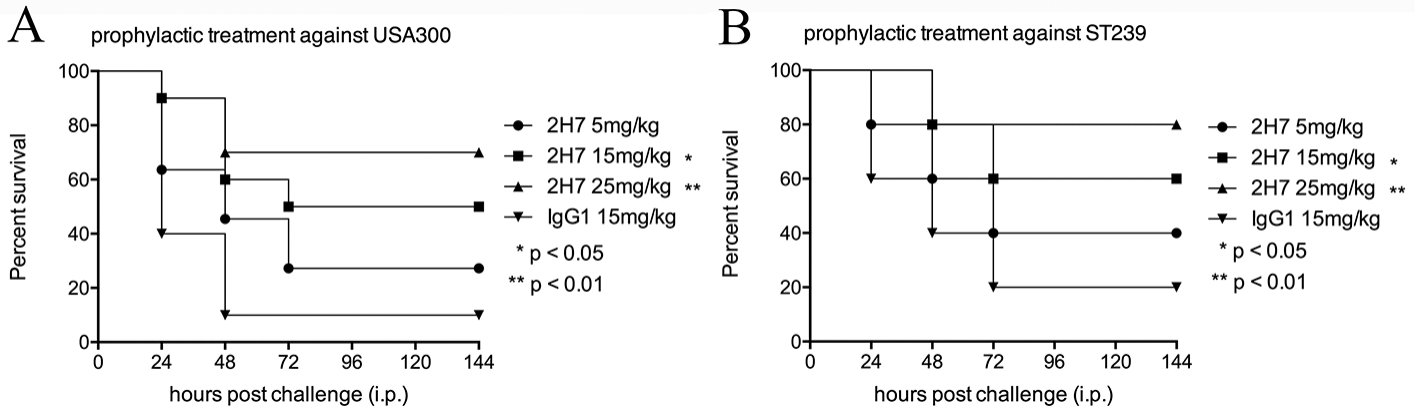
Prophylactic administration of 2H7 protects mice against lethal MRSA challenge in a peritonitis model. Female BALB/c mice (n = 10) were challenged with 2×10^9^ CFUs of USA300 (A) or 4×10^9^ CFUs of ST239 (B) by intraperitoneal injection. Mice were treated intraperitoneally with a single dose (at 5, 15 or 25 mg/kg) of 2H7 or isotype control mAb (15 mg/kg) 24 h before infection. The challenged mice were monitored over a period of 144 h. The significance of the protection was analyzed by the log-rank Mantel-Cox test (**: p<0.01, *: p<0.05). i.p., intraperitoneal.

MRSA peritonitis imposes great risks on patients undergoing peritoneal dialysis [30]. We investigated whether 2H7 could protect mice in a peritoneal infection model. BALB/c mice (n = 10) were treated intraperitoneally with a single dose (at 5, 15 or 25 mg/kg) of 2H7 or isotype control mAb IgG1 (15 mg/kg). Twenty-four hours following passive immunization, challenges with 2×10^9^ CFUs of USA300 were administered intraperitoneally. Most of the control antibody-treated mice (~90%) did not survive the challenge with USA300 within the first 48 h (Fig 6A). In contrast, 2H7 prophylaxis (at 15 mg/kg or 25 mg/kg) resulted in a significant increase in survival rate compared with isotype control antibody (15 mg/kg, the survival rate is 50%, p = 0.015; 25 mg/kg, the survival rate is 70%, p = 0.0041) (Fig 6A). In addition, 2H7 prophylaxis (at 15 mg/kg or 25 mg/kg) was able to confer significant protection against lethal intraperitoneal challenge with 4×10^9^ CFUs of ST239 (15 mg/kg, the survival rate is 60%, p = 0.034; 25 mg/kg, the survival rate is 80%, p = 0.0067) (Fig 6B). However, therapeutic administration (1 h or 3 h post challenge) of 2H7 didn’t confer protective efficacy in the peritoneal challenge model (data not shown).

### Treatment with 2H7 provides protection against staphylococcal disease

In the murine peritoneal infection model, *S*. *aureus* peritonitis is associated with formation of abscess lesions on the abdominal walls [8]. BALB/c mice (n = 10) were passively immunized with 2H7 (15 mg/kg) or isotype control mAb IgG1 (15 mg/kg) 24 h prior to intraperitoneal challenge with 1×10^9^ CFUs of USA300. On day 2 following challenge, the mice were euthanized by CO_2_ inhalation, necropsied, and examined for abscesses on the abdominal wall. As described in earlier studies [8], most control antibody-treated mice harbored yellow staphylococcal lesions on the abdominal wall at the injection site. Our results showed that significantly less numbers of mice presenting with peritoneal abscess lesions were found in mice received 2H7 rather than mice received IgG1 control antibody (p = 0.0255) (Table 1).

**Table 1:** 2H7 prevents the formation of *S*. *aureus* peritoneal abscesses.

mAb^a^	Number of mice presenting with peritoneal abscess lesions^b^	Number of mice presenting without peritoneal abscess lesions	p^c^
IgG1	9	1	
2H7	3	7	0.0255

^*a*^ BALB/c mice (female, 6-week-old, n = 10) were injected intraperitoneally with a single dose (15 mg/kg) of 2H7 or isotype control mAb 24 h prior to intraperitoneal challenge with 1×10^9^ CFUs of USA300.

^*b*^ On day 2 following intraperitoneal challenge, the mice were euthanized by CO_2_ inhalation and examined for the formation of peritoneal abscesses on the abdominal wall.

^c^ The formation of peritoneal abscesses was analyzed through the Chi-square with Yates' correction test (two-sided).

**Fig 7:**
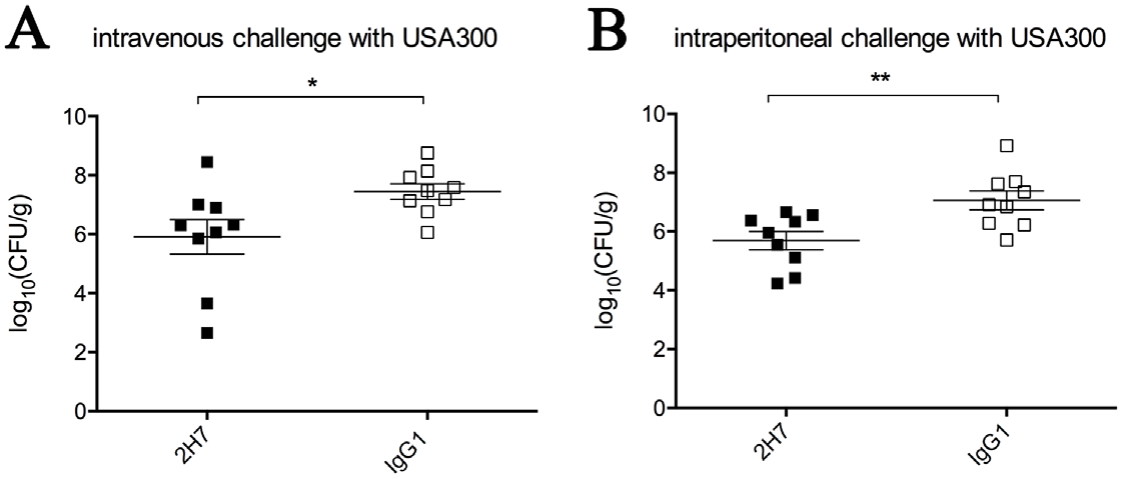
Treatment with 2H7 reduces the staphylococcal burden in kidneys. BALB/c mice (female, 6-week-old, n = 9) were injected intraperitoneally with a single dose (15 mg/kg) of 2H7 or isotype control mAb 24 h prior to challenge. On day 2 following intraperitoneal challenge and day 4 following intravenous challenge, the infected mice were euthanized by CO_2_ inhalation. The staphylococcal loads in the both kidneys were measured by plating the homogenized renal tissue on TSA plates. (A) Mice were challenged intravenously with 1×10^7^ CFUs of USA300. (B) Mice were challenged intraperitoneally with 1×10^9^ CFUs of USA300. The data represent the log_10_ (CFU/g) means ± SEM. The statistical significance was analyzed with a two-tailed unpaired t test (*: p<0.05, **: p<0.01).

Kidneys are the major infection organs after intravenous and intraperitoneal challenges with *S*. *aureus* [14,31]. To examine if 2H7 enhance bacterial clearance in the murine model, BALB/c mice (n = 9) were passively immunized with 2H7 (15 mg/kg) or isotype control mAb IgG1 (15 mg/kg) 24 h prior to challenge by intravenous injection with 1×10^7^ CFUs of USA300 (Fig 7A) or by intraperitoneal injection with 1×10^9^ CFUs of USA300 (Fig 7B). On day 2 following intraperitoneal challenge and day 4 following intravenous challenge, the infected mice were euthanized by CO_2_ inhalation, and both kidneys were homogenized. In the intravenous challenge model, staphylococcal loads of 7.45±0.26 log10 CFU/g were isolated from the renal homogenates of control mice. Compared with mice treated with isotype mAb IgG1, the mice that received 2H7 displayed a reduction of 1.54±0.64 (p = 0.0294) log10 CFU/g in the staphylococcal burden (Fig 7A). In the intraperitoneal challenge model, renal homogenates of control mice revealed staphylococcal loads of 7.06±0.32 log10 CFU/g. Significant staphylococcal burden reduction was also observed in the mice received 2H7 (1.38±0.44, p = 0.0069) (Fig 7B).

## Discussion

MRSA, a bacterial that resistant to multiple antibiotics, is considered as a “superbug” and is responsible for several life-threatening infections in humans. In an era of MRSA epidemic and lack of new classes of antibiotics, immunotherapeutic approaches directed against *S*. *aureus* have been broadly investigated [5]. MAbs are attractive antibody-based therapeutic reagents because of their homogeneity, stability, low-immunogenicity and amenability for engineering to improve various preferred characteristics [32]. Therefore, mAbs are well poised to be important candidates in a new age of antimicrobial therapy [33]. To the best of our knowledge, we provide the first evidence that an anti-SasA mAb is effective for the prevention and treatment of MRSA infections in murine models. Considering that SasA is prevalent and the NRR region of SasA is conserved among clinical *S*. *aureus* strains, SasA is a promising target for mAb-based immunotherapy against MRSA infections.

Compared with other cell wall-associated proteins of *S*. *aureus*, such as iron-regulated surface determinant B (IsdB) (~72 kDa), Staphylococcal protein A (SPA) (~50 kDa), clumping factor A (ClfA) (~110 kDa) and fibronectin-binding protein A (FnbpA) (~112 kDa), SasA is a relatively large surface-exposed protein (~240 kDa). It is difficult to construct full-length recombinant SasA. Based on the sequence analysis (the overall structure, sequence conservation and immunoprotective domain), we selected the SRR1-NRR1 (48–540 aa) region of SasA as the immunogen and generated mAb 2H7. The data showed that the binding epitope of 2H7 is within the NRR1 region (230–540 aa) and 2H7 can recognize the conserved NRR region of SasA. The structure of the NRR region was recently determined, revealing that the N terminus of NRR (245–492 aa) is extended outward by a relatively rigid structure of the C terminus of NRR (492–751 aa) [34]. Thus, NRR1 (230–540 aa) is likely to be located within the surface-exposed region of SasA, which is consistent with the result that 2H7 is able to bind native SasA expressed on the surface of *S*. *aureus*. It has been reported that SasA can mediate the binding of *S*. *aureus* to platelets [20]. However, 2H7 is not capable of inhibiting the binding of SasA to mouse platelets, suggesting that 2H7 may not provide protection in a staphylococcal endocarditis model [20]. In line with previously reported mAbs targeting surface proteins of *S*. *aureus* (immunodominant staphylococcal antigen A (IsaA) [14], IsdB [12] and SPA [15]), 2H7 can enhance the opsonophagocytic killing of *S*. *aureus* by both mouse and human neutrophils.

We evaluated the protective efficacy of 2H7 in both a murine sepsis model and a murine peritoneal infection model. These models mimic staphylococcal infections of end-stage renal disease (ESRD) patients undergoing dialysis. Chronic dialysis patients are vulnerable to MRSA infections [35,36]. It is estimated that the prevalence of MRSA colonization is 7.2% in hemodialysis patients and 1.3% in patients undergoing peritoneal dialysis [35]. Notably, colonized patients have a much higher risk of developing MRSA infections than noncolonized patients [35]. In agreement with an earlier report [8], in our study, a large number of *S*. *aureus* (2–4×10^9^ CFU) were required to develop lethal disease in the intraperitoneal challenge model. In contrast, intravenous injection of 1–2×10^8^ CFU of *S*. *aureus* was lethal in mice.

In the sepsis model, prophylactic administration of 2H7 at a dose of 15 mg/kg was sufficient to confer protection against USA300 and ST239 challenge, with a protection rate of 50% and 60%, respectively, compared with 90%-100% mortality in the control group. This protective efficacy is comparable to results obtained from prophylactic treatment with mAbs targeting IsdB [16], ClfA [11], IsaA [17] and alpha-hemolysin [14] [9]. Only a few studies evaluated the therapeutic potential of mAbs in murine models for *S*. *aureus* sepsis. It has been reported that an mAb to IsdB provided protection when dosed 1 h prior to challenge but is unable to confer protection when dosed 1 h post challenge [12]. In our study, therapeutic administration (1 h post challenge) of 2H7 significantly improved the survival of mice, despite the fact that the survival rate was lower than that obtained from prophylactic administration. However, the therapeutic window can’t be extended beyond 3 h, which is consistent with a previous report [17]. In the peritoneal infection model, prophylactic administration of 2H7 at a dose of 15 mg/kg was sufficient to confer protection against lethal challenges with both USA300 and ST239. Earlier work indicated that alpha-Hemolysin is an important virulence factor in the peritoneal infection model [8]. However, passive immunization of an Hla-neutralizing mAb didn’t prevent the formation of abscess lesions in the peritoneal cavity [8]. In our study, 2H7 prophylaxis was able to offer protection against staphylococcal abscess lesions in the peritoneal infection model. Of note, in both the sepsis model and the peritoneal infection model, passive immunization of 2H7 can reduce the staphylococcal loads in kidneys.

We don’t expect that an anti-SasA mAb can be the single component of an immunotherapy that designs to combat against MRSA infections in all conditions. *S*. *aureus* harbors divergent virulence factors for tissue adhesion, evasion of host immune defense and cell lysis [37]. For example, Alpha-Hemolysin is a pore-forming cytotoxin of *S*. *aureus* and Hla-neutralizing mAbs mediate protection against *S*. *aureus* pneumonia, peritonitis, sepsis and skin abscess [7–9]. SpA enables *S*. *aureus* to resist host immune responses and anti-SpA mAbs prevent *S*. *aureus* disease in mice [15]. The variance and redundancy of *S*. *aureus* virulence factors call for immunotherapy against multiple antigens [5,6,38]. Passive immunization with combined antibodies, such as the combination of a SasA-specific mAb, an anti-SpA mAb and an Hla-neutralizing mAb, is likely to provide enhanced protective efficacy against MRSA infections.

In summary, this work demonstrated that passive immunization with 2H7 was able to confer protection against murine sepsis and peritonitis caused by USA300 and ST239, which are prevalent MRSA clones in North America and Asian countries, respectively. The protective efficacy of 2H7 indicates that SasA is a novel candidate target for a mAb-based immunotherapy against MRSA infections. Further study of the therapeutic activity of a humanized version of 2H7 and its possible synergistic effect with antibiotics or other mAbs will help to relate this immunotherapy strategy to the clinical context.

## Supporting Information

S1 Fig The phylogenetic tree of 19 different sequence types of *S*. *aureus* strains used in bioinformatic analysis of SasA. The concatenated sequence was generated from 7 housekeeping genes used in multilocus sequence typing (MLST) of *S*. *aureus*, yielding 3,186 aligned nucleotide positions. The phylogenetic tree was built by Neighbour-joining method. The numbers next to each node display substitutions per site and tip labels refer to the multilocus sequence type of *S*. *aureus*.
(TIF)

S2 Fig Immunization with SRR1 and NRR1 region of SasA generated protective immunity against lethal infection with *S*. *aureus*. Female BALB/c mice (6-week-old, n = 9) were immunized with 20 μg of SRR1, NRR, NRR1, NRR2 or PBS formulated with aluminum hydroxide adjuvant. Booster immunizations were performed 14 and 28 d after the initial vaccination. Seven days following the last booster immunization (day 35), the mice were infected via intraperitoneal injection with 2 x 10^9^ CFUs of USA300. The challenged mice were monitored for survival over a period of 120 h. The significance of the protection was analyzed by the log-rank Mantel-Cox test. *: p<0.05.
(TIF)

S1 Table Primers used in this study. (DOCX)

S2 Table Completed *Staphylococcus aureus* genomes and SasA homology. (DOCX)

S3 Table ELISA titer for mAbs binding to SasA fragments. (DOCX)

S4 Table Passive immunization with anti-SasA mAbs and protection against *S*. *aureus* USA300 in a murine intraperitoneal challenge model. (DOCX)
