# Supplementary figures and images for: Monoclonal Antibody Targeting Staphylococcus aureus Surface Protein A (SasA) Protect Against Staphylococcus aureus Sepsis and Peritonitis in Mice
Source: PLoS One. 2016 Feb 29;11(2):e0149460. doi: 10.1371/journal.pone.0149460 (PMC4771200; doi:10.1371/journal.pone.0149460)

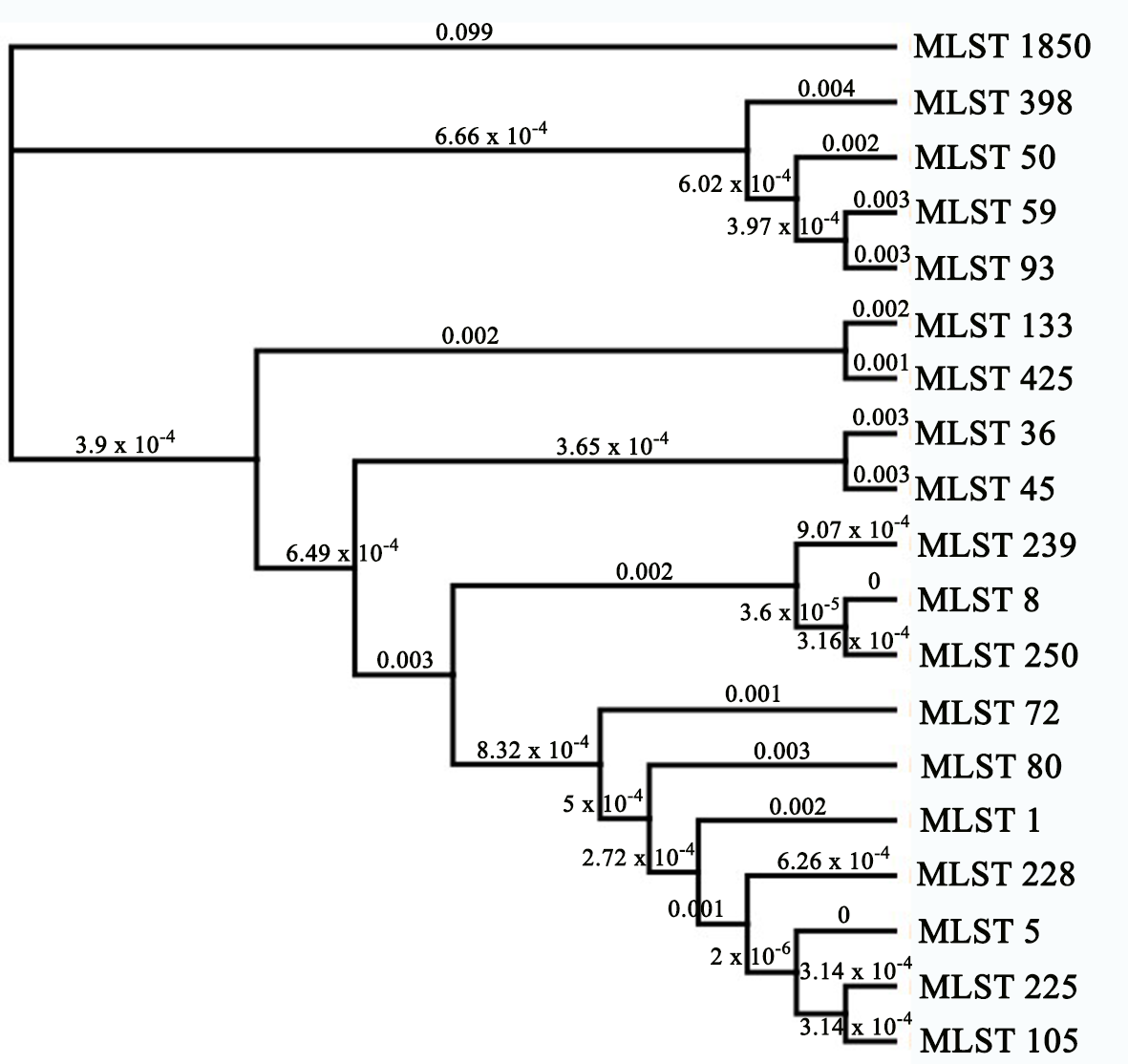

Supplement: S1 Fig — The concatenated sequence was generated from 7 housekeeping genes used in multilocus sequence typing (MLST) of S. aureus, yielding 3,186 aligned nucleotide positions. The phylogenetic tree was built by Neighbour-joining method. The numbers next to each node display substitutions per site and tip labels refer to the multilocus sequence type of S. aureus. (TIF) [file pone.0149460.s001.tif]

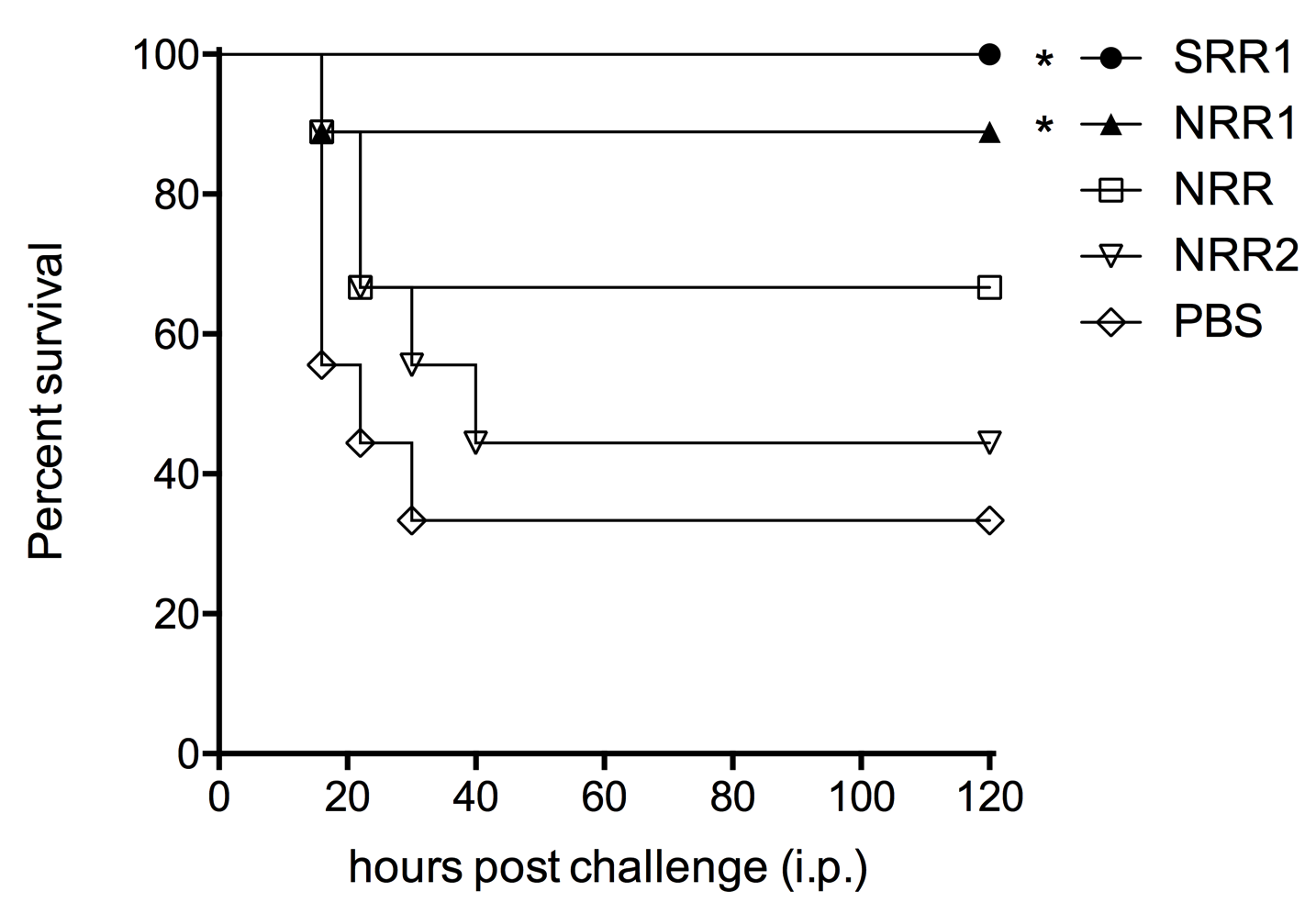

Supplement: S2 Fig — Female BALB/c mice (6-week-old, n = 9) were immunized with 20 μg of SRR1, NRR, NRR1, NRR2 or PBS formulated with aluminum hydroxide adjuvant. Booster immunizations were performed 14 and 28 d after the initial vaccination. Seven days following the last booster immunization (day 35), the mice were infected via intraperitoneal injection with 2 x 10^9^ CFUs of USA300. The challenged mice were monitored for survival over a period of 120 h. The significance of the protection was analyzed by the log-rank Mantel-Cox test. *: p<0.05. (TIF) [file pone.0149460.s002.tif]
