# Supplementary material for: Monoclonal Antibody Targeting Staphylococcus aureus Surface Protein A (SasA) Protect Against Staphylococcus aureus Sepsis and Peritonitis in Mice
Source: PLoS One. 2016 Feb 29;11(2):e0149460. doi: 10.1371/journal.pone.0149460 (PMC4771200; doi:10.1371/journal.pone.0149460)
Supplement: S1 Table — (DOCX) [file pone.0149460.s003.docx]

**Table S1. Primers used in this study**

| Fragments of SasA | Forward primer (5’-3’) | Reverse primer (5’-3’) |
| --- | --- | --- |
| SRR1 | CGCATATGAGTCATAGTTTAGTGAGTCAAG | TACTCGAGGGTGCTAGTTGACGTTGT |
| NRR | ATGCTAGCACCGCACCAGTAAAACTT | CGCTCGAGATTTCTTGTTACTTCATATT |
| NRR1 | CGCATATGGCACCAGTAAAACTTCGAACTT | CGCTCGAGGTAGTTATATCCTTTAGC |
| NRR2 | ATGCTAGCGAATATACAGAGTCTGCTGTT | CGCTCGAGATTTCTTGTTACTTCATATTT |
| SRR1-NRR1 | CGCATATGAGTCATAGTTTAGTGAGTCAAG | CGCTCGAGGTAGTTATATCCTTTAGC |
