# Supplementary material for: Monoclonal Antibody Targeting Staphylococcus aureus Surface Protein A (SasA) Protect Against Staphylococcus aureus Sepsis and Peritonitis in Mice
Source: PLoS One. 2016 Feb 29;11(2):e0149460. doi: 10.1371/journal.pone.0149460 (PMC4771200; doi:10.1371/journal.pone.0149460)
Supplement: S2 Table — (DOCX) [file pone.0149460.s004.docx]

**Table S2. Completed *Staphylococcus aureus* genomes and SasA homology**

| Strain | Reference number | Features | Isolation date | Origin | MLST | % SRR1 identitya | % NRR identityb | % SRR2 identityc |
| --- | --- | --- | --- | --- | --- | --- | --- | --- |
| MW2 [1] | NC_003923 | CA-MRSA | 1998 | USA | 1 | 100.0 | 99.0 | 98.7 |
| MSSA476 [2] | NC_002953 | CA-MSSA | 1998 | UK | 1 | 100.0 | 99.0 | 98.6 |
| ED98 [3] | CP001781 | Poultry strain | 1996-1997 | Ireland | 5 | 100.0 | 99.8 | 98.4 |
| Mu3 [4] | NC_009782 | HA-VISA | 1997 | Japan | 5 | 100.0 | 99.8 | 98.4 |
| Mu50 [5] | NC_002758 | HA-VISA | 1997 | Japan | 5 | 100.0 | 99.8 | 98.4 |
| N315 [5] | NC_002745 | HA-MRSA | 1982 | Japan | 5 | 100.0 | 99.8 | 98.4 |
| VC40 [6] | NC_016912 | Laboratory VRSA | before 2012 | Germany | 8 | 100.0 | 100.0 | 99.9 |
| USA300 TCH1516 [7] | NC_010079 | CA-MRSA | before 2004 | USA | 8 | 100.0 | 100.0 | 100 |
| Newman [8] | AP009351 | MSSA | 1952 | UK | 8 | 100.0 | 100.0 | 99.9 |
| USA300 FPR3757 [9] | CP000255 | CA-MRSA | before 2002 | USA | 8 | - | - | - |
| NCTC8325 [10] | NC_007795 | Laboratory strain | before 1949 | UK | 8 | 100.0 | 100.0 | 99.9 |
| M1 [11] | NC_021059 | MRSA | 2003 | Denmark | 8 | 100.0 | 100.0 | 95.3 |
| USA500 2395 [12] | CP007499 | MRSA | 1996 | USA | 8 | 100.0 | 99.8 | 95.7 |
| MRSA 252 [2] | BX571856 | HA-MRSA | 1997 | UK | 36 | 92.9 | 98.5 | 81 |
| CA-347 [13] | NC_021554 | MRSA | 2005 | USA | 45 | 97.8 | 97.7 | 92.2 |
| 6850 [14] | NC_022222 | MSSA | before 1987 | USA | 50 | 97.8 | 98.5 | 88.6 |
| M013 [15] | NC_016928 | CA-MRSA | 2002 | Taiwan | 59 | 97.8 | 98.5 | 90.1 |
| SA40 [16] | NC_022443 | CA-MRSA | 2005 | Taiwan | 59 | 97.8 | 98.5 | 90.1 |
| SA957 [16] | NC_022442 | CA-MRSA | 2000 | Taiwan | 59 | 97.8 | 98.5 | 90.1 |
| CN1 [17] | NC_022226 | CA-MRSA | 2007 | Korea | 72 | 100 | 99.8 | 96.2 |
| 11819-97 [18] | NC_017351 | CA-MRSA | before 2012 | Denmark | 80 | 100.0 | 100.0 | 93.8 |
| JKD6159 [19] | NC_017338 | CA-MRSA | 2004 | Australia | 93 | 97.8 | 99 | 85.4 |
| JH9 [20] | NC_009487 | HA-VISA | 2000 | USA | 105 | 100.0 | 99.8 | 98.4 |
| JH1 [20] | NC_009632 | HA-VISA | 2000 | USA | 105 | 100.0 | 99.8 | 98.4 |
| ED133 [21] | NC_017337 | Ovine mastitis strain | 1997 | France | 133 | 96.7 | 97.7 | 82.2 |
| 04-02981 [22] | NC_017340 | HA-MRSA | 2004 | Germany | 225 | 100.0 | 99.8 | 98.4 |
| ST228 18583 [23] | NC_020568 | MRSA | 2008 | Switzerland | 228 | 100.0 | 99.8 | 98.4 |
| T0131 [24] | NC_017347 | HA-MRSA | 2006 | China | 239 | 92.9 | 98.5 | 86.5 |
| TW20 [25] | NC_017331 | HA-MRSA | 2003 | UK | 239 | 92.9 | 98.5 | 86.5 |
| JKD6008 [25] | NC_017341 | HA-VISA | 2003 | New Zealand | 239 | 92.9 | 98.5 | 86.5 |
| BMB9393 [26] | CP005288 | MRSA | 1993 | Brazil | 239 | 92.9 | 98.5 | 86.5 |
| Z172 [27] | CP006838 | HA-MRSA | 2010 | Taiwan | 239 | 92.9 | 98.5 | 86.5 |
| COL [28] | CP000046 | MRSA | 1961 | UK | 250 | 100.0 | 100.0 | 98.4 |
| 08BA02176 [29] | NC_018608 | LA-MRSA | 2008 | Canada | 398 | 92.3 | 96.9 | 92 |
| LGA251 [30] | NC_017349 | Cattle MRSA | 2007 | UK | 425 | 97.8 | 98.3 | 75.8 |
| MSHR1132 [31] | NC_016941 | CA-MRSA | 2006 | Australia | 1850 | 85.7 | 94.8 | 81.1 |

MLST, multi-locus sequence type;

a The SRR1 sequence (Amino acid) identity between USA300 FPR3757 and other *S.aureus* strains

b The NRR sequence identity (Amino acid)between USA300 FPR3757 and other *S.aureus* strains

c The SRR2 sequence (Amino acid) identity between USA300 FPR3757 and other *S.aureus* strains

**References**

1. Baba T, Takeuchi F, Kuroda M, Yuzawa H, Aoki K, et al. (2002) Genome and virulence determinants of high virulence community-acquired MRSA. Lancet 359: 1819-1827.

2. Holden MT, Feil EJ, Lindsay JA, Peacock SJ, Day NP, et al. (2004) Complete genomes of two clinical Staphylococcus aureus strains: evidence for the rapid evolution of virulence and drug resistance. Proc Natl Acad Sci U S A 101: 9786-9791.

3. Lowder BV, Guinane CM, Ben Zakour NL, Weinert LA, Conway-Morris A, et al. (2009) Recent human-to-poultry host jump, adaptation, and pandemic spread of Staphylococcus aureus. Proc Natl Acad Sci U S A 106: 19545-19550.

4. Neoh HM, Cui L, Yuzawa H, Takeuchi F, Matsuo M, et al. (2008) Mutated response regulator graR is responsible for phenotypic conversion of Staphylococcus aureus from heterogeneous vancomycin-intermediate resistance to vancomycin-intermediate resistance. Antimicrob Agents Chemother 52: 45-53.

5. Kuroda M, Ohta T, Uchiyama I, Baba T, Yuzawa H, et al. (2001) Whole genome sequencing of meticillin-resistant Staphylococcus aureus. Lancet 357: 1225-1240.

6. Sass P, Berscheid A, Jansen A, Oedenkoven M, Szekat C, et al. (2012) Genome sequence of Staphylococcus aureus VC40, a vancomycin- and daptomycin-resistant strain, to study the genetics of development of resistance to currently applied last-resort antibiotics. J Bacteriol 194: 2107-2108.

7. Highlander SK, Hulten KG, Qin X, Jiang H, Yerrapragada S, et al. (2007) Subtle genetic changes enhance virulence of methicillin resistant and sensitive Staphylococcus aureus. BMC Microbiol 7: 99.

8. Baba T, Bae T, Schneewind O, Takeuchi F, Hiramatsu K (2008) Genome sequence of Staphylococcus aureus strain Newman and comparative analysis of staphylococcal genomes: polymorphism and evolution of two major pathogenicity islands. J Bacteriol 190: 300-310.

9. Diep BA, Gill SR, Chang RF, Phan TH, Chen JH, et al. (2006) Complete genome sequence of USA300, an epidemic clone of community-acquired meticillin-resistant Staphylococcus aureus. Lancet 367: 731-739.

10. Berscheid A, Sass P, Weber-Lassalle K, Cheung AL, Bierbaum G (2012) Revisiting the genomes of the Staphylococcus aureus strains NCTC 8325 and RN4220. Int J Med Microbiol 302: 84-87.

11. Larner-Svensson H, Worning P, Bartels MD, Hestbjerg Hansen L, Boye K, et al. (2013) Complete Genome Sequence of Staphylococcus aureus Strain M1, a Unique t024-ST8-IVa Danish Methicillin-Resistant S. aureus Clone. Genome Announc 1.

12. Benson MA, Ohneck EA, Ryan C, Alonzo F, 3rd, Smith H, et al. (2014) Evolution of hypervirulence by a MRSA clone through acquisition of a transposable element. Mol Microbiol 93: 664-681.

13. Stegger M, Driebe EM, Roe C, Lemmer D, Bowers JR, et al. (2013) Genome Sequence of Staphylococcus aureus Strain CA-347, a USA600 Methicillin-Resistant Isolate. Genome Announc 1.

14. Fraunholz M, Bernhardt J, Schuldes J, Daniel R, Hecker M, et al. (2013) Complete Genome Sequence of Staphylococcus aureus 6850, a Highly Cytotoxic and Clinically Virulent Methicillin-Sensitive Strain with Distant Relatedness to Prototype Strains. Genome Announc 1.

15. Huang TW, Chen FJ, Miu WC, Liao TL, Lin AC, et al. (2012) Complete genome sequence of Staphylococcus aureus M013, a pvl-positive, ST59-SCCmec type V strain isolated in Taiwan. J Bacteriol 194: 1256-1257.

16. Chen CJ, Unger C, Hoffmann W, Lindsay JA, Huang YC, et al. (2013) Characterization and comparison of 2 distinct epidemic community-associated methicillin-resistant Staphylococcus aureus clones of ST59 lineage. PLoS One 8: e63210.

17. Chen Y, Chatterjee SS, Porcella SF, Yu YS, Otto M (2013) Complete genome sequence of a Panton-Valentine leukocidin-negative community-associated methicillin-resistant Staphylococcus aureus strain of sequence type 72 from Korea. PLoS One 8: e72803.

18. Stegger M, Price LB, Larsen AR, Gillece JD, Waters AE, et al. (2012) Genome sequence of Staphylococcus aureus strain 11819-97, an ST80-IV European community-acquired methicillin-resistant isolate. J Bacteriol 194: 1625-1626.

19. Chua K, Seemann T, Harrison PF, Davies JK, Coutts SJ, et al. (2010) Complete genome sequence of Staphylococcus aureus strain JKD6159, a unique Australian clone of ST93-IV community methicillin-resistant Staphylococcus aureus. J Bacteriol 192: 5556-5557.

20. Mwangi MM, Wu SW, Zhou Y, Sieradzki K, de Lencastre H, et al. (2007) Tracking the in vivo evolution of multidrug resistance in Staphylococcus aureus by whole-genome sequencing. Proc Natl Acad Sci U S A 104: 9451-9456.

21. Guinane CM, Ben Zakour NL, Tormo-Mas MA, Weinert LA, Lowder BV, et al. (2010) Evolutionary genomics of Staphylococcus aureus reveals insights into the origin and molecular basis of ruminant host adaptation. Genome Biol Evol 2: 454-466.

22. Nubel U, Dordel J, Kurt K, Strommenger B, Westh H, et al. (2010) A timescale for evolution, population expansion, and spatial spread of an emerging clone of methicillin-resistant Staphylococcus aureus. PLoS Pathog 6: e1000855.

23. Vogel V, Falquet L, Calderon-Copete SP, Basset P, Blanc DS (2012) Short term evolution of a highly transmissible methicillin-resistant Staphylococcus aureus clone (ST228) in a tertiary care hospital. PLoS One 7: e38969.

24. Li Y, Cao B, Zhang Y, Zhou J, Yang B, et al. (2011) Complete genome sequence of Staphylococcus aureus T0131, an ST239-MRSA-SCCmec type III clone isolated in China. J Bacteriol 193: 3411-3412.

25. Holden MT, Lindsay JA, Corton C, Quail MA, Cockfield JD, et al. (2010) Genome sequence of a recently emerged, highly transmissible, multi-antibiotic- and antiseptic-resistant variant of methicillin-resistant Staphylococcus aureus, sequence type 239 (TW). J Bacteriol 192: 888-892.

26. Costa MO, Beltrame CO, Ferreira FA, Botelho AM, Lima NC, et al. (2013) Complete Genome Sequence of a Variant of the Methicillin-Resistant Staphylococcus aureus ST239 Lineage, Strain BMB9393, Displaying Superior Ability To Accumulate ica-Independent Biofilm. Genome Announc 1.

27. Chen FJ, Lauderdale TL, Wang LS, Huang IW (2013) Complete Genome Sequence of Staphylococcus aureus Z172, a Vancomycin-Intermediate and Daptomycin-Nonsusceptible Methicillin-Resistant Strain Isolated in Taiwan. Genome Announc 1.

28. Gill SR, Fouts DE, Archer GL, Mongodin EF, Deboy RT, et al. (2005) Insights on evolution of virulence and resistance from the complete genome analysis of an early methicillin-resistant Staphylococcus aureus strain and a biofilm-producing methicillin-resistant Staphylococcus epidermidis strain. J Bacteriol 187: 2426-2438.

29. Golding GR, Bryden L, Levett PN, McDonald RR, Wong A, et al. (2012) whole-genome sequence of livestock-associated st398 methicillin-resistant staphylococcus aureus Isolated from Humans in Canada. J Bacteriol 194: 6627-6628.

30. Garcia-Alvarez L, Holden MT, Lindsay H, Webb CR, Brown DF, et al. (2011) Meticillin-resistant Staphylococcus aureus with a novel mecA homologue in human and bovine populations in the UK and Denmark: a descriptive study. Lancet Infect Dis 11: 595-603.

31. Holt DC, Holden MT, Tong SY, Castillo-Ramirez S, Clarke L, et al. (2011) A very early-branching Staphylococcus aureus lineage lacking the carotenoid pigment staphyloxanthin. Genome Biol Evol 3: 881-895.
