# Supplementary material for: Monoclonal Antibody Targeting Staphylococcus aureus Surface Protein A (SasA) Protect Against Staphylococcus aureus Sepsis and Peritonitis in Mice
Source: PLoS One. 2016 Feb 29;11(2):e0149460. doi: 10.1371/journal.pone.0149460 (PMC4771200; doi:10.1371/journal.pone.0149460)
Supplement: S3 Table — (DOCX) [file pone.0149460.s005.docx]

**Table S3. ELISA titer for mAb binding to SasA fragments**

| mAb | ELISA titer^a^ to antigen or antigen fragment | | | |
| --- | --- | --- | --- | --- |
|  | SRR1-NRR1 | SRR1 | NRR1 | NRR2 |
| #2 | 256,000 | 128,000 | <500 | <500 |
| #5 | 128,000 | 64,000 | 1,000 | <500 |
| #11 | 64,000 | <500 | 8,000 | <500 |
| #23 | 256,000 | <500 | <500 | <500 |
| 1E7 | 128,000 | <500 | 128,000 | <500 |
| 6E7 | 128,000 | <500 | 128,000 | <500 |
| 2H7 | 256,000 | <500 | 256,000 | <500 |

^a^ The antibody-positive cut-off values were set as two times greater than the OD450 means of the blank control. An ELISA antibody titer was expressed as the highest serum dilution giving a positive reaction.
