# Supplementary material for: Monoclonal Antibody Targeting Staphylococcus aureus Surface Protein A (SasA) Protect Against Staphylococcus aureus Sepsis and Peritonitis in Mice
Source: PLoS One. 2016 Feb 29;11(2):e0149460. doi: 10.1371/journal.pone.0149460 (PMC4771200; doi:10.1371/journal.pone.0149460)
Supplement: S4 Table — (DOCX) [file pone.0149460.s006.docx]

**Table S4. Passive immunization with anti-SasA mAbs and protection against *S.aureus* USA300 in a murine intraperitoneal challenge model**

| mAb | Number of mice surviving^a^ | | | | | | Total (%) | P ^b^ |
| --- | --- | --- | --- | --- | --- | --- | --- | --- |
|  | Hours post challenge | | | | | |  |  |
|  | 0h | 24h | 48h | 72h | 96h | 120h |  |  |
| IgG1 |  | | | | | | | |
| Control ^c^ | 10 | 5 | 2 | 1 | 1 | 1 | 1(10%) |  |
| #2 | 10 | 6 | 4 | 2 | 2 | 2 | 2(20%) | 0.433 |
| #5 | 10 | 4 | 2 | 0 | 0 | 0 | 0(0%) | 0.738 |
| 1E7 | 10 | 5 | 2 | 0 | 0 | 0 | 0(0%) | 0.649 |
| 6E7 | 10 | 7 | 4 | 2 | 2 | 2 | 2(20%) | 0.339 |
| 2H7 | 10 | 8 | 6 | 6 | 6 | 6 | 6(60%) | 0.024 |
| IgG2b |  | | | | | | | |
| Control ^d^ | 10 | 6 | 3 | 1 | 1 | 1 | 1(10%) |  |
| #11 | 10 | 5 | 3 | 1 | 1 | 1 | 1(10%) | 0.885 |
| #23 | 10 | 7 | 5 | 3 | 3 | 3 | 3(30%) | 0.290 |

^a^ Female BALB/c mice (6-week-old, n=10) were injected intraperitoneally with a single dose of anti-SasA mAb or isotype control mAb (15mg/kg) 24 h prior to challenge by intraperitoneal injection with 2×10^9^ CFUs of USA300. The challenged mice were monitored for survival over a period of 120 h

^b^ The survival curves were analyzed by the log-rank Mantel-Cox test.

^c^ isotype control mAb IgG1

^d^ isotype control mAb IgG2b
